# Supplementary material for: Limb work and joint work minimization reveal an energetic benefit to the elbows-back, knees-forward limb design in parasagittal quadrupeds
Source: Proc Biol Sci. 2020 Dec 9;287(1940):20201517. doi: 10.1098/rspb.2020.1517 (PMC7739919; doi:10.1098/rspb.2020.1517)
Supplement: Supplementary Table and Figure and coding details [file rspb20201517supp1.docx]

**Supplementary Material for:**

**Limb work and joint work minimization reveal an energetic benefit to the elbows-back, knees-forward limb design in parasagittal quadrupeds.**

James R. Usherwood and Michael C. Granatosky

Proceedings of the Royal Society, B

Doi: 10.1098/rspb.2020.1517

Code and data available on Dryad:

https://doi.org/10.5061/dryad.76hdr7sv4

**Supplemental Table S1: Data (mean ± standard deviation) on the timing of peak vertical force as a percentage of stance phase for the forelimb and hindlimb of forty quadrupedal tetrapods.**

| Species | Number of individuals | Limb | Number of steps | Timing of peak vertical force (% of stance phase) | Original source |
| --- | --- | --- | --- | --- | --- |
| *Ambystoma mexicanum* | 4 | FL | 12 | 56.70 ± 13.53 | 1 |
|  |  | HL | 29 | 44.88 ± 13.46 |  |
| *Ambystoma tigrinum* | 5 | FL | 0 | - | 2 |
|  |  | HL | 116 | 27.34 ± 16.19 |  |
| *Aotus nancymaae* | 3 | FL | 13 | 52.45 ± 21.51 | 3 |
|  |  | HL | 16 | 45.55 ± 16.11 |  |
| *Ateles fusciceps* | 1 | FL | 12 | 67.43 ± 12.58 | 4 |
|  |  | HL | 10 | 33.66 ± 13.49 |  |
| *Ateles geoffroyi* | 1 | FL | 8 | 52.76 ± 3.24 | 4 |
|  |  | HL | 15 | 45.27 ± 4.39 |  |
| *Caiman crocodilus* | 2 | FL | 8 | 67.42 ± 8.95 | 1 |
|  |  | HL | 12 | 32.23 ± 12.57 |  |
| *Caracal caracal* | 2 | FL | 20 | 43.14 ± 16.77 | 4 |
|  |  | HL | 17 | 29.15 ± 10.49 |  |
| *Cebus capucinus* | 3 | FL | 15 | 53.64 ± 18.71 | 5 |
|  |  | HL | 14 | 37.72 ± 16.76 |  |
| *Daubentonia madagascariensis* | 3 | FL | 46 | 57.37 ± 15.84 | 4 |
|  |  | HL | 28 | 28.20 ± 4.57 |  |
| *Desmodus rotundus* | 5 | FL | 23 | 52.84 ± 16.61 | 6 |
|  |  | HL | 14 | 40.52 ± 19.85 |  |
| *Didelphis virginiana* | 5 | FL | 48 | 70.89 ± 8.85 | 7 |
|  |  | HL | 52 | 32.44 ± 15.33 |  |
| *Erythrocebus patas* | 1 | FL | 3 | 46.49 ± 15.54 | 4 |
|  |  | HL | 8 | 40.78 ± 3.78 |  |
| *Eulemur fulvus* | 2 | FL | 8 | 44.86 ± 13.92 | 7 |
|  |  | HL | 6 | 31.29 ± 9.42 |  |
| *Felis catus* | 2 | FL | 14 | 40.07 ± 11.54 | 4 |
|  |  | HL | 13 | 25.39 ± 6.51 |  |
| *Hapalemur griseus* | 2 | FL | 20 | 53.99 ± 13.71 | 4 |
|  |  | HL | 13 | 32.18 ± 9.83 |  |
| *Iguana iguana* | 2 | FL | 8 | 62.77 ± 6.25 | 1 |
|  |  | HL | 7 | 29.70 ± 9.45 |  |
| *Leiocephalus schreibersii* | 2 | FL | 17 | 45.91 ± 12.04 | 8 |
|  |  | HL | 14 | 38.56 ± 7.02 |  |
| *Lemur catta* | 3 | FL | 28 | 57.04 ± 9.93 | 9 |
|  |  | HL | 25 | 31.04 ± 8.66 |  |
| *Leopardus pardalis* | 1 | FL | 14 | 35.29 ± 14.05 | 4 |
|  |  | HL | 12 | 24.96 ± 10.32 |  |
| *Leptailurus serval* | 2 | FL | 16 | 45.75 ± 17.75 | 4 |
|  |  | HL | 15 | 19.04 ± 1.93 |  |
| *Macaca fascicularis* | 2 | FL | 19 | 55.18 ± 4.08 | 4 |
|  |  | HL | 14 | 44.72 ± 4.82 |  |
| *Macaca mulatta* | 2 | FL | 9 | 55.31 ± 8.54 | 4 |
|  |  | HL | 12 | 38.48 ± 4.82 |  |
| *Nasua narica* | 2 | FL | 57 | 44.11 ± 13.71 | 7 |
|  |  | HL | 61 | 33.15 ± 11.07 |  |
| *Oplurus cuvieri* | 3 | FL | 19 | 50.27 ± 9.84 | 8 |
|  |  | HL | 17 | 47.98 ± 9.18 |  |
| *Panthera tigris* | 4 | FL | 43 | 45.00 ± 21.02 | 4 |
|  |  | HL | 38 | 26.87 ± 16.58 |  |
| *Papio anubis* | 2 | FL | 4 | 50.15 ± 11.31 | 4 |
|  |  | HL | 4 | 43.99 ± 4.11 |  |
| *Pleurodeles waltl* | 3 | FL | 4 | 51.22 ± 19.79 | 1 |
|  |  | HL | 3 | 42.32 ± 25.18 |  |
| *Potos flavus* | 2 | FL | 150 | 54.24 ± 7.59 | 7 |
|  |  | HL | 158 | 33.74 ± 11.19 |  |
| *Propithecus coquereli* | 3 | FL | 14 | 45.60 ± 10.68 | 10 |
|  |  | HL | 15 | 41.91 ± 7.49 |  |
| *Pseudemys concinna* | 3 | FL | 0 | - | 11 |
|  |  | HL | 52 | 39.02 ± 8.57 |  |
| *Saimiri sciureus* | 6 | FL | 79 | 49.14 ± 14.16 | 4 |
|  |  | HL | 52 | 33.70 ± 12.62 |  |
| *Salvator merianae* | 3 | FL | 0 | - | 12 |
|  |  | HL | 56 | 11.92 ± 12.15 |  |
| *Smaug warreni* | 2 | FL | 10 | 52.70 ± 10.23 | 8 |
|  |  | HL | 11 | 53.71 ± 13.61 |  |
| *Stellagama stellio* | 2 | FL | 11 | 43.56 ± 6.64 | 8 |
|  |  | HL | 11 | 50.93 ± 10.95 |  |
| *Testudo graeca* | 4 | FL | 34 | 60.28 ± 8.25 | 7 |
|  |  | HL | 37 | 39.29 ± 7.20 |  |
| *Testudo horsfieldii* | 1 | FL | 25 | 65.56 ± 17.34 | 7 |
|  |  | HL | 38 | 32.22 ± 12.97 |  |
| *Tiliqua scincoides* | 2 | FL | 9 | 59.52 ± 9.15 | 13 |
|  |  | HL | 8 | 35.05 ± 24.22 |  |
| *Tropidurus torquatus* | 2 | FL | 7 | 51.50 ± 10.46 | 8 |
|  |  | HL | 6 | 44.57 ± 36.18 |  |
| *Varanus exanthematicus* | 2 | FL | 10 | 49.66 ± 6.47 | 8 |
|  |  | HL | 10 | 52.35 ± 28.44 |  |
| *Varecia variegata* | 3 | FL | 40 | 51.88 ± 7.11 | 9 |
|  |  | HL | 31 | 27.55 ± 5.76 |  |

1. Nyakatura JA *et al.* 2019 Reverse-engineering the locomotion of a stem amniote. *Nature* **565**, 351. (doi:10.1038/s41586-018-0851-2)

2. Sheffield KM, Blob RW. 2011 Loading mechanics of the femur in tiger salamanders (Ambystoma tigrinum) during terrestrial locomotion. *Journal of Experimental Biology* **214**, 2603–2615.

3. Hanna JB, Granatosky MC, Rana P, Schmitt D. 2017 The evolution of vertical climbing in primates: evidence from reaction forces. *Journal of Experimental Biology* **220**, 3039–3052. (doi:10.1242/jeb.157628)

4. Granatosky MC, Fitzsimons A, Zeininger A, Schmitt D. 2018 Mechanisms for the functional differentiation of the propulsive and braking roles of the forelimbs and hindlimbs during quadrupedal walking in primates and felines. *Journal of Experimental Biology* **221**, 1–11. (doi:10.1242/jeb.162917)

5. Granatosky MC, Schmitt D. 2019 The mechanical origins of arm-swinging. *Journal of Human Evolution* **130**, 61–71. (doi:10.1016/j.jhevol.2019.02.001)

6. Granatosky MC. 2018 Forelimb and hindlimb loading patterns during quadrupedal locomotion in the large flying fox (Pteropus vampyrus) and common vampire bat (Desmodus rotundus). *Journal of Zoology* **305**, 63–72. (doi:10.1111/jzo.12538)

7. Granatosky MC *et al.* 2020 Variation in limb loading magnitude and timing in tetrapods. *J. Exp. Biol.* **223**. (doi:10.1242/jeb.201525)

8. McElroy EJ, Wilson R, Biknevicius AR, Reilly SM. 2014 A comparative study of single-leg ground reaction forces in running lizards. *Journal of Experimental Biology* **217**, 735–742. (doi:10.1242/jeb.095620)

9. Granatosky MC, Tripp CH, Schmitt D. 2016 Gait kinetics of above and below branch quadrupedal locomotion in lemurid primates. *Journal of Experimental Biology* **219**, 53–63.

10. Granatosky MC, Tripp CH, Fabre A-C, Schmitt D. 2016 Patterns of quadrupedal locomotion in a vertical clinging and leaping primate (Propithecus coquereli) with implications for understanding the functional demands of primate quadrupedal locomotion. *Am. J. Phys. Anthropol.* **160**, 644–652. (doi:10.1002/ajpa.22991)

11. Butcher MT, Blob RW. 2008 Mechanics of limb bone loading during terrestrial locomotion in river cooter turtles (Pseudemys concinna). *Journal of Experimental Biology* **211**, 1186–1186.

12. Sheffield KM, Butcher MT, Shugart SK, Gander JC, Blob RW. 2011 Locomotor loading mechanics in the hindlimbs of tegu lizards (Tupinambis merianae): comparative and evolutionary implications. *Journal of Experimental Biology* **214**, 2616–2630. (doi:10.1242/jeb.048801)

13. Nyakatura JA, Andrada E, Curth S, Fischer MS. 2014 Bridging “Romer’s Gap”: Limb Mechanics of an Extant Belly-Dragging Lizard Inform Debate on Tetrapod Locomotion During the Early Carboniferous. *Evol Biol* **41**, 175–190. (doi:10.1007/s11692-013-9266-z)


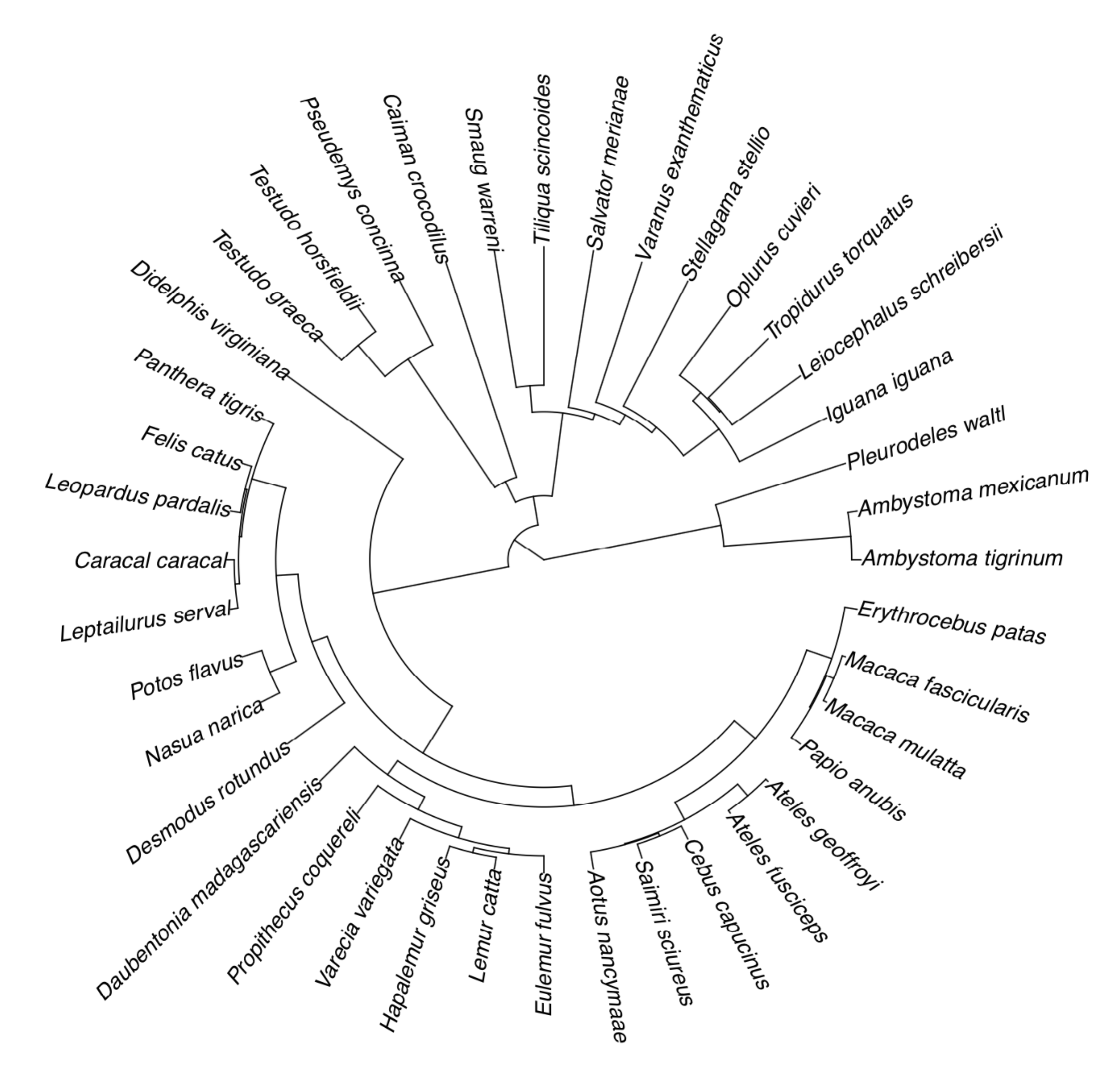


**Supplemental Figure S1: Phylogeny of species used in this study.** Tree topology and the depth of each node w as based off the recent supertree reported at [www.timetree.org](http://www.timetree.org) (Kumar et al., 2017).

**Further details of coding and simulation.**

**See Dryad: https://doi.org/10.5061/dryad.76hdr7sv4**

Simulations were written in National Instruments LabVIEW 2012. Powers are deliberately reported as arbitrary units as the principles are not dependent on specific force, length or timing magnitudes. Code is presented as ESM_Simulation1, ESM_Simulation2. Simulation outputs in ESM_outputs_Sim1, ESM_ouputs_Sim2

*Details for Figure 1 (Simulation 1)*

Symmetrical ‘Bouncing’ kinetics and centre of mass kinematics were derived through stance (horizontal: *S*_x,t_; vertical *S*_y,t_) through numerical integration (1000 counts over a nominal 0.125 second stance *T*_stance_; dt=0.000125s). The initial (and so also final) centre of mass height was 0.85m meaning that, with 0.5m proximal and distal segments, the leg was slightly flexed at beginning and end of stance. The initial vertical velocity *U*_y_ conditions were derived from ballistics with an aerial phase *T*_ballistic_ of 0.125s to result in a symmetrical stance:

$U_{\text{y}}=-g\frac{T_{\text{ballistic}}}{2}$.

The (mass specific) vertical force through stance was modeled as a half sine-wave:

$$F_{\text{y,t}}=A_{\text{Fy}}\sin\left(\frac{\pi t}{T_{\text{stance}}}\right)$$

with an amplitude *A*_Fy_ sufficient to result in weight support over the stride:

$$A_{\text{Fy}}=\pi g\frac{\left(T_{\text{ballistic}}+T_{\text{stance}}\right)}{2T_{\text{stance}}}$$

With these initial conditions of height and vertical velocity, vertical position though stance were calculated through integration.

In terms of centre of mass trajectory, fluctuations in horizontal velocity were neglected, meaning that the horizontal position about the foot origin for bouncing mean horizontal velocity *V*_x_ (taken as 8m/s) through time *t* was

$S_{\text{x,t}}=\frac{-V_{\text{x}}T_{\text{stance}}}{2}+V_{\text{x}}t$ .

At each instant, the angle between vertical and the centre of mass-foot line was calculated as

 .

The (mass specific) horizontal force through time was that which resulted in a resultant force producing the vertical force derived above, orientated at a constant proportion *p* between vertical and centre of mass:

 .

We focus on the purely vertical (*p*=0) and purely axial (*p*=1); intermediate values of *p* give intermediate results.

The resulting position of the centre of mass (treated as the proximal joint) at each instant resulted in two potential configurations: intermediate joint forward or backward (below). Moments about the proximal and intermediate joints were calculated through time given their positions and the ground reaction force. The changing geometry provided the angular velocities of each joint, and so the joint powers (the product of moment and angular velocity).

*Details for Figure 4 (Simulation 2)*

Forces and geometries were calculated for 100 divisions of stance (with arbitrary time interval). Limb geometries were calculated for a limb of two even segment lengths from fully outstretched at the beginning of stance to fully outstretched, and with horizontal translation of the foot. Vertical force profiles were calculated following Jayes and Alexander, 1980, resulting in continuous weight support without imposing roll or pitch moments. This is consistent with a horizontal translation of the centre of mass, and means that no limb work is required. The geometries assumed for this were that: step length = step width (between left and right) = 1/3 back length (between hips and shoulders).

Jayes AS, Alexander RM. The gaits of chelonians: walking techniques for very low speeds. 1980. Journal of Zoology 191:353–78. doi: 10.1111/j.1469-7998.1980.tb01464.x

**Further ESM on Dryad**

Coordinates for Figure 2 are given in ESM_Coords_Fig2.xls
